# Supplementary material for: Prevalence of insomnia and associated factors among postpartum mothers in Southern Ethiopia, a community-based cross-sectional study
Source: PLoS One. 2024 Jul 23;19(7):e0307104. doi: 10.1371/journal.pone.0307104 (PMC11265656; doi:10.1371/journal.pone.0307104)
Supplement: Attachment — Submitted filename: Response to Reviewers.docx [file pone.0307104.s002.docx]

**Reviewer 1**

**Dear reviewer**, thank you for your valuable comments and suggestions. It was very helpful. We have tried to address all the comments and suggestions on the main manuscript. Here, we have to clarify some important issues.

Thank you again for your time.

**Comment**

- In the abstract, consider one sentence distinguishing insomnia in postpartum from sleep disruption in postpartum due to direct childcare.

**Response**: Thank you for the comment. We have amended it in the manuscript.

- I am not sure about the final sentence of the abstract. It feels strange to me to strongly advise a planned index pregnancy. On what grounds is this recommendation made? Consider amending this language. Correlation does not equate causation.

**Response**: Thank you for the comment. We have amended it in the manuscript.

- P.7 the formatting and capitalization of academic affiliations is not consistent (ex. 1 Department of Midwifery, 2Department of midwifery).

**Response**: Thank you for the comment. We have amended it in the manuscript.

- Introduction- I would consider a different definition of insomnia. The important distinction between postpartum insomnia and postpartum sleep disruption is- is the person having difficulty sleeping, outside of direct childcare. Basically, when given the opportunity, still struggling with their sleep..

**Response**: Thank you for the comment. The definition of insomnia is the same during the postpartum period when mothers are obliged for childcare with those having no such obligation. The tool (Athens Insomnia Scale) that we have used to measure insomnia during the postpartum period is also used for the general population. The issue is that women have added burden of insomnia due to many reasons including childcare during postpartum period.

- Consider removing this sentence or clarifying what is meant by ‘atypical’- Women have a 40% higher risk and an atypical presentation of insomnia than men (3, 6).

**Response**: Thank you for the comment. We have amended it in the manuscript.

- P. 14 might be nice to include monthly income converted to USD or another currency so more readers can comprehend.

**Response**: We appreciate your comment, the value of the local currency (Ethiopian birr (ETB)) when changed to USD or other currencies varies within a short period of time. As a result the value of ETB during the time of data collection would be difficult to gather.

- P. 18- consider ‘met criteria for insomnia’ rather than ‘had insomnia’

**Response**: Thank you for the comment. We have amended it in the manuscript.

- P. 22 “This might be due to the definite link between depression and insomnia, as well as insomnia, being a manifestation of depression (30).” Consider amending the language to acknowledge the bi directional relationship for example “This finding may be explained by the well-established and bi directional relationship between insomnia and depression.

**Response**: Thank you for the comment. We have amended it in the manuscript.

- For the conclusion- consider mentioning established implications of insomnia in postpartum

**Response**: Thank you for the comment. We have amended it in the manuscript.

- Please consider amending this sentence, it is hard to understand “As a result, the sustainable development goals for mental health can be achieved; even if it appears that world leaders are now off-track in achieving mental health targets, and hence, the following recommendations were made for actions to be performed by the stakeholders”

**Response**: Thank you for the comment. We have amended it in the manuscript

**Reviewer 2**

Dear reviewer, thank you for your valuable comments and suggestions. It was very helpful. We really appreciate it. Here, we have to clarify the issue.

**Comment:** I was curious about your definition of chronic insomnia - you mention difficulty sleeping that lasts for greater than 3 weeks. The definition I am more familiar with indicates chronicity begins after 3 months - was this a different definition specifically for post-partum patients?

**Response:** Thank you for the comment. What we have written is not the definition of chronic insomnia. It is the definition of long-term insomnia. We have classified insomnia into transient, short-term and long-term (not acute and chronic). To our knowledge too chronic insomnia is insomnia lasting more than 3 months.
